# Leaf morphological traits show greater responses to changes in climate than leaf physiological traits and gas exchange variables

**DOI:** 10.1002/ece3.10941

**Published:** 2024-03-19

**Authors:** Susan E. Everingham, Catherine A. Offord, Manon E. B. Sabot, Angela T. Moles

**Affiliations:** ^1^ Evolution and Ecology Research Centre, School of Biological, Earth and Environmental Sciences UNSW Sydney New South Wales Australia; ^2^ The Australian Institute of Botanical Science, The Australian PlantBank, Royal Botanic Gardens and Domain Trust, Australian Botanic Garden Mount Annan Mount Annan New South Wales Australia; ^3^ Institute of Plant Sciences University of Bern Bern Switzerland; ^4^ Oeschger Centre for Climate Change Research University of Bern Bern Switzerland; ^5^ Climate Change Research Centre UNSW Sydney New South Wales Australia; ^6^ Australian Research Council Centre of Excellence for Climate Extremes UNSW Sydney New South Wales Australia

**Keywords:** climate change, leaf morphology, leaf shape, LMA, photosynthesis, plant traits

## Abstract

Adaptation to changing conditions is one of the strategies plants may use to survive in the face of climate change. We aimed to determine whether plants' leaf morphological and physiological traits/gas exchange variables have changed in response to recent, anthropogenic climate change. We grew seedlings from resurrected historic seeds from ex‐situ seed banks and paired modern seeds in a common‐garden experiment. Species pairs were collected from regions that had undergone differing levels of climate change using an emerging framework—Climate Contrast Resurrection Ecology, allowing us to hypothesise that regions with greater changes in climate (including temperature, precipitation, climate variability and climatic extremes) would be greater trait responses in leaf morphology and physiology over time. Our study found that in regions where there were greater changes in climate, there were greater changes in average leaf area, leaf margin complexity, leaf thickness and leaf intrinsic water use efficiency. Changes in leaf roundness, photosynthetic rate, stomatal density and the leaf economic strategy of our species were not correlated with changes in climate. Our results show that leaves do have the ability to respond to changes in climate, however, there are greater inherited responses in morphological leaf traits than in physiological traits/variables and greater responses to extreme measures of climate than gradual changes in climatic means. It is vital for accurate predictions of species' responses to impending climate change to ensure that future climate change ecology studies utilise knowledge about the difference in both leaf trait and gas exchange responses and the climate variables that they respond to.

## Graphical abstract

In regions in Australia where there were great climate changes, plant species showed greater changes in average leaf area, leaf margin complexity, leaf thickness and leaf intrinsic water use efficiency. These results were found using a climate contrast resurrection ecology method comparing historic seedlings grown from seeds banked in ex‐situ seedbanks and modern seedlings collected from seeds in the field both grown in a common environment. There were greater responses in morphological leaf traits than in physiological leaf traits and variables. Leaf traits and variables are responding more strongly to extreme measures of climate than gradual changes in climatic means.

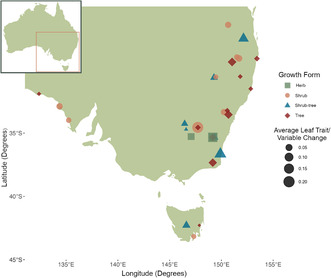

## 1 INTRODUCTION

Unprecedented climate changes have resulted in a heightened risk of extinction for many plant species (Hughes, 2000; Parmesan, 2006; Walther et al., 2002). However, plants can respond morphologically and physiologically to changes in their environment, which may allow them to adapt to continuing climate change (Ahrens et al., 2019; Everingham et al., 2021; Nicotra et al., 2010). Leaf traits, for example, vary inter‐ and intra‐specifically across biogeographic gradients (Gallagher & Leishman, 2012a; Moles et al., 2014; Wright et al., 2005) and have changed in response to temperature and precipitation in experimental manipulations (Henn et al., 2018; Liancourt et al., 2015; Nicotra et al., 2008). A lack of historical data for many traits and variables and for species in some regions of the world has made it difficult to determine whether the degree to which within‐species leaf traits and gas exchange variables have changed in response to recent anthropogenic climate change in wild plant populations. For example, photosynthetic rates were not often measured before 1990 due to technological limitations. Similarly, there is limited historical data for traits that are difficult or expensive to measure (such as nitrogen content, but see Chibnall, 1923; McHargue & Roy, 1932), particularly for non‐agricultural species or species outside North America or Europe. To address this gap, we used a recently developed framework—‘Climate Contrast Resurrection Ecology’ (Everingham et al., 2021) to determine whether plant leaf traits and photosynthetic variables have responded to recent anthropogenic climate change in their natural habitats.

The Climate Contrast Resurrection Ecology framework (Everingham et al., 2021) overcomes absent or limited historic data by measuring traits on matched resurrected historic seeds (Franks et al., 2017; Weider et al., 2017). However, rather than look at the absolute amount of trait difference between the historic and modern seeds (which is likely affected by viability losses in the historic seeds and/or selection during seedling emergence and establishment), our method uses the fact that climate change has not been evenly distributed across the landscape (Everingham et al., 2021). South‐eastern Australia provides a key example of this uneven change as temperature and precipitation, along with climate change variables, have increased and decreased to different extents during the last 30–40 years (Australian Bureau of Meteorology, 2019). The Climate Contrast Resurrection Ecology framework calculates whether species from regions with larger climate changes also have increased leaf trait change over time (Everingham et al., 2021). This method counteracts the issues of seed storage, maternal effects and intraspecific trait variability impacting trait changes through time as these may affect the elevation of the regression between climate change and trait change but will not affect the slope of this relationship (Everingham et al., 2021). In this study, we used this Climate Contrast Resurrection Ecology framework to test a series of hypotheses about how leaf morphological traits (including leaf size, shape, margin complexity and thickness) and physiological traits and gas exchange variables (including photosynthetic rate, stomatal density, intrinsic water use efficiency (iWUE) and leaf economic strategy) have responded to changes in climate.

Our first hypothesis was that leaf area would have increased to a greater extent in regions with greater increases in temperature and/or precipitation. Leaf area typically increases with increasing temperature (where precipitation permits, however, in hot dry climates, leaf areas are predominately smaller than those in more mesic conditions, Wright et al., 2017) and/or precipitation and this is evident across large geographic scales, in global meta‐analyses across species (Gallagher & Leishman, 2012b; Moles et al., 2014) and palaeobotanical studies within species (Ng & Smith, 2020). However, some studies based on herbarium specimens show no change in leaf area within a species, through time, with increases in mean annual precipitation (Li et al., 2019) and in regions with extremely high temperatures, inter‐specific studies have shown species tend to have smaller leaves to conserve energy through reduced evaporative cooling (Wright et al., 2004). Likewise, at regional and local scales, within‐species studies tend to find no significant relationship between leaf area and precipitation (Guittar et al., 2016; Ordoñez et al., 2010). Leaf area is a key trait that affects plant growth, survival and reproduction (Wang et al., 2019) and determining how leaf area is changing in response to climate change will help us to understand species' differing responses to climate change.

Second, we hypothesised that leaves would have increased in elongation (i.e. decrease in width‐to‐length ratio) in regions with increasing temperature and/or decreasing precipitation. Longer, narrower leaves decrease the boundary layer of the leaf surface without the need for a reduction in overall leaf size, enabling plants to shed heat through sensible heat loss rather than transpiration cooling whilst maintaining the optimum surface area for light capture. Narrower leaves might also allow for high transpiration when evaporative demand is low and precipitation is high, which may lead to increased photosynthesis and nutrient uptake (Yates et al., 2009). Previous work shows that along large biogeographic gradients, across multiple species, leaves tend to become more elongated with increasing temperatures (Radice & Arena, 2014; Traiser et al., 2005) and decreasing precipitation (Jacobs, 1999; Traiser et al., 2005; but see Radice & Arena, 2014; Xu et al., 2009). Herbarium data show that some species' leaves are becoming longer and narrower with increasing temperature and precipitation over time (Li et al., 2019). However, contrasting data show that within some species, leaf length decreased over time with increasing maximum and minimum temperatures (Leger, 2013). Although this does suggest that leaf roundness can change in response to climate over long time frames, it is not yet clear whether plants will be able to change their leaf roundness quickly enough to keep pace with the recent climate changes and our study aimed to address this knowledge gap.

Next, we predicted that leaf margin complexity (the ratio of leaf area to leaf perimeter) would have increased in regions with decreasing temperature and/or increasing precipitation. Leaves may have more complex margins in cooler and/or higher moisture environments due to the gas‐exchange hypothesis which states that species in cooler environments have more complex margins or increased lobing/leaf teeth as these leaf margin sites are spaces of increased gas exchange earlier in the growing season when there are lower temperatures but higher moisture and nutrient availability (Royer & Wilf, 2006). This relationship has been found in previous studies measuring leaf toothedness (Royer & Wilf, 2006) and this may be correlated with margin complexity as measured in this study. However, leaf thermal studies have shown that margin complexity is weakly related to leaf temperature or leaf thermal dynamics (Leigh et al., 2017). With decreasing precipitation, plants should respond by lowering their margin complexity to reduce water loss from transpiration. Although it has not been measured in the field historically, leaf margin complexity has been shown to increase in cooler regions in intraspecific palaeobotanical records and across climate gradients (Njoku, 1957; Peppe et al., 2011; Royer, 2012; Royer et al., 2009). There is far less evidence for relationships between leaf margin complexity and mean precipitation and our study aims to add to our knowledge by quantifying this response.

We hypothesised that species would have increased leaf thickness in regions with increases in temperature and/or decreases in precipitation. Increased leaf thickness protects species from heat damage (Groom et al., 2004) and leads to greater leaf thermal mass and therefore slower thermal responses to changes in temperature (Curtis et al., 2012; Leigh et al., 2012). Increased leaf thickness leads to decreases in water loss per unit volume (compared to leaves of the same size and stomatal resistance) which is adaptive for leaves when higher temperatures or lower rainfall has led to increased leaf transpiration (Chitwood & Sinha, 2016; Groom et al., 2004). Increases in leaf thickness are also associated with decreased air spaces in leaves and dry matter per volume, resulting in raised leaf thermal capacity, which could also favour increased leaf thickness under increasing temperatures or decreasing precipitation (Groom et al., 2004; Roderick et al., 1999). Meta‐analyses and biogeographical patterns across species, as well as long‐term experimental manipulations within species typically find that leaf thickness tends to increase with increasing temperature and decreasing precipitation (Groom et al., 2004; Niinemets, 2001; Schollert et al., 2015). Determining if leaves are getting thicker through time in response to recent climate change is important for understanding how carbon capture and plant‐herbivore relationships may change in the future.

Photosynthetic traits and gas exchange rates have rarely been quantified historically due to technological limitations and this limits our knowledge of real‐world shifts in gas exchange rates and water use efficiency in response to anthropogenic climate change. We hypothesised that in regions with increases in temperature and/or precipitation, stomatal density and photosynthetic rates would increase and iWUE would decrease. Temperature increases may result in increased enzyme activity and photosynthetic rates and decreases in iWUE, which is evident in single‐species experimental manipulations (Thomas et al., 2007), short‐term field observations (Slot & Winter, 2017) and modelling studies (Guo et al., 2010). However, some manipulative experiments have shown no apparent changes in photosynthesis when temperatures are artificially increased (Quentin et al., 2015; Song et al., 2016), while others have shown decreases in photosynthesis when temperatures are increased, particularly for cold‐climate adapted species (Drake et al., 2015) and some have shown increases in iWUE with increasing temperature (Fajardo et al., 2019). Decreases in precipitation might favour species with higher iWUE to maximise photosynthesis and therefore decrease water loss when water is limited. Stomatal density typically increases with temperature up to a temperature threshold point in manipulative experiments (Yan et al., 2017; but see Beerling & Chaloner, 1993). Increases in mean temperature experienced in the last three to four decades (but not past a high‐temperature threshold) lead to increases in the photosynthetic enzymatic function and this may lead to species increasing in stomatal density to maximise photosynthetic rates. Decreases in precipitation may lead to species reducing stomatal density to reduce transpiration and this has been found in geographical gradients (Hogan et al., 1994; Schoettle & Rochelle, 2000 but see Hill et al., 2015). Measuring species' responses to recent anthropogenic climate change is an important supplement to the current experimental and geographical gradient data in determining species' abilities to respond physiologically to climate change.

Finally, we considered leaf economic responses to changes in climate. Leaf physiology and structure exist on a spectrum across most species, communities, soil types and ecosystems (Reich & Flores‐Moreno, 2017; Wright et al., 2004). Globally, climate variables are not the primary predictors or drivers of leaf economic traits such as leaf mass per unit area (LMA) or leaf nitrogen content and LMA has been found to be more strongly explained by trait coordination and soil types (Wright et al., 2004; but see Sastry & Barua, 2017 where increases in LMA led to increased leaf thermotolerance under extreme temperatures). There is also evidence that the coordination between leaf nitrogen content and other plant traits can be explained by short‐term climate (i.e. a few weeks) and plant optimality theory (Caldararu et al., 2020). However, within‐species studies at the site level have shown that increases in LMA are related to increases in temperature (Gallagher & Leishman, 2012b; Moles et al., 2014; Swenson et al., 2012) and precipitation (Moles et al., 2014). We hypothesised that species may move towards a ‘faster’ leaf economic strategy with increasing temperature and/or precipitation i.e. LMA would decrease while photosynthetic rate and leaf nitrogen content would increase. On the other hand, in regions with decreasing precipitation, plants may experience increased transpiration rates and would need to adopt a slower leaf economic strategy to avoid water‐loss damage.

Although it is vital to determine recent plant responses to mean climate metrics, (i.e. mean temperature and mean precipitation as per our predictions above), changes in extreme measures of climate and increased climate variability may also affect plant traits/variables (Katz & Brown, 1992; Reyer et al., 2013; Yue et al., 2019). We hypothesised that changes in extreme measures of climate and climatic variability (including changes in heatwave duration, dry spell duration, drought duration and changes in climate range and variability) would have been more strongly related to changes in leaf traits/variables than changes in mean climate. Increases in extreme climate events may induce a ‘threshold’ response where leaf traits/variables exhibit extreme responses when pushed too far beyond typical climate envelopes for longer periods (Yue et al., 2019). Similarly, plants may have been responding more rapidly to changes in climatic variability or climatic range (Katz & Brown, 1992; Reyer et al., 2013). Disentangling responses in leaf traits/gas exchange variables to mean changes in climate from changes to extreme measures of climate is vital in determining future species' survival under predicted climate change scenarios.

## 2 MATERIALS AND METHODS

### 2.1 Seeds and seedlings

**FIGURE 1:**
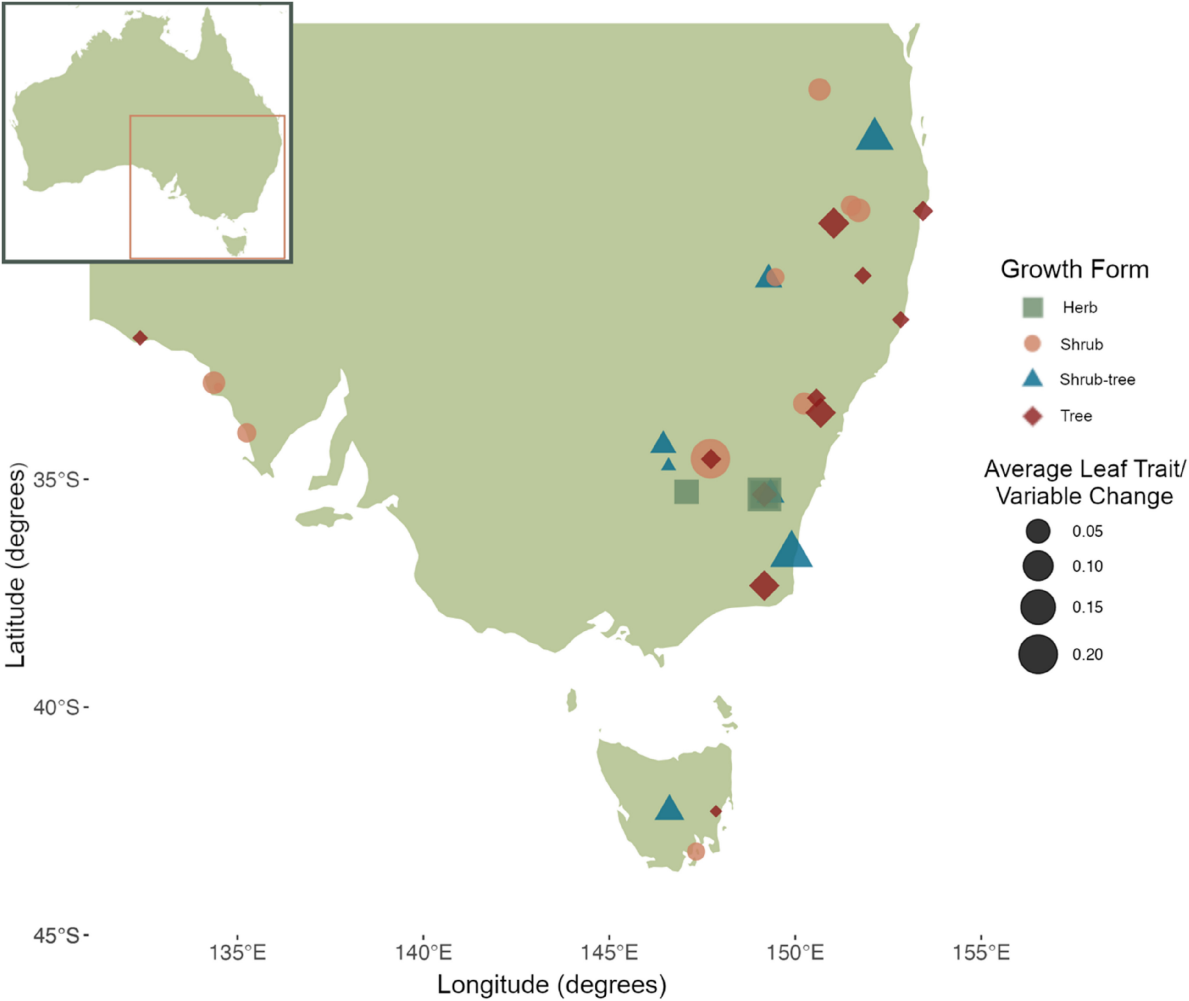
Locations of populations where each species' historic and modern seeds were collected in southeastern Australia—a single point represents a single species. Points are colour‐ and shape‐coded by their growth‐form and the size of the points are scaled to represent the overall amount of leaf trait and variable change for any given species across all traits. This overall change for each species was calculated as the absolute value of the average across all changes (log‐transformed ratio of means between the historic and modern populations) for all traits and variables.

Historic seeds were acquired for 32 species from stored collections in ex‐situ seed banks at The Australian PlantBank and the Australian National Botanic Garden. This included four herbaceous species, ten shrubs, seven shrub trees and eleven trees where all shrubs, shrub‐trees and trees were evergreen (Figure 1; See Appendix S1: Table A1 for full species list, as well as the growth form and leaf characteristics, i.e. compound or simple, of each species). Due to the nature of Australian native species and Australian ecology (predominantly evergreen species and very limited deciduous plants Orians & Milewski, 2007), all 32 species were evergreen. Matched modern seeds from the same species as the historic seeds were collected in the same location, at the same time of year as their historic counterparts (Figure 1; details on collection methods and location data in Everingham et al., 2021 and Appendix S1: Table A1). The amount of time between the historic and modern seed collections ranged from 29 to 40 years.

Seeds were germinated on water agar (0.7% w.v.) in controlled incubators. Most species were germinated at 20°C with a 12‐h light, 12‐h dark cycle, but some species required specific germination treatments such as gibberellic acid (GA_3_), smoke water (1%) or specific temperature and light treatments (see Appendix S1: Section A1, Table A2 for full germination treatment methods). Treatments were always kept constant for modern and historic seeds of each species. After germination, we transferred up to 50 germinated seeds to trays made up of 24‐cells each measuring 4 cm (depth) by 2 cm^2^ (square area) cells. The seedlings grew for 2 weeks in the trays to ensure early seedling survival before being transferred to individual 1.9 L pots. Potting soil comprised of 33% Australian Native Landscape supply of ‘Organic Garden Mix’, 33% washed river sand and 33% Cocopeat as well as a general slow‐release fertiliser added at 200 mL per 75 L of soil. Plants were grown in a glasshouse at UNSW, Sydney for 6 months with an overhead irrigation system. Pots were randomised each month to reduce position effects.

### 2.2 Plant trait and gas exchange variable measurements

After the 6‐month growing period, we measured a range of morphological leaf traits including leaf area, leaf roundness, leaf margin complexity and leaf thickness following standard protocols (Pérez‐Harguindeguy et al., 2013). Full details on the morphological traits measured and the protocols used can be found in Appendix S1: Section A2.

We measured physiological variables including leaf photosynthetic rate, iWUE and leaf nitrogen content. To obtain photosynthetic measurements, we used portable infrared gas analysers (LICOR 6400XT, Lincoln, Nebraska) on well‐watered, non‐root‐bound, non‐flowering individuals. We randomly selected a subset of ten historic plants and ten modern plants from each species. Some species had fewer than ten plants available and some species were excluded from photosynthetic measurements because their leaves were not large enough to fit into the gas chamber without damage to the majority of the seedlings (see Appendix S1: Table A3 for sample sizes). We took infrared gas measurements on the youngest fully expanded mature leaf following standard protocols (Evans & Santiago, 2014) between the hours of 10:00 to 14:00 (Australian Eastern Standard Time) on days with no visible cloud cover. We ensured that for each species, infrared gas exchange measurements were taken on historic and modern plants at random within 30 min to minimise changes in light or temperature. Our measurements were made under constant saturating light conditions (1800 μmol m^−2^ s^−1^) provided by a constant light source in the LICOR chamber. The chamber CO_2_ concentration was set at 400 ppm and the temperature was set at 25°C. We took five consecutive measurements approximately 2 s apart and used the average of these five measurements. We recorded the light‐saturated photosynthetic rate (*A*_sat_; μmol CO_2_ m^−2^ s^−1^) and the stomatal conductance (*g*_s_; mol H_2_O m^−2^ s^−1^) and then calculated iWUE as the ratio between photosynthetic rate and stomatal conductance.

To quantify leaf nitrogen, we harvested leaves at 6 months, dried them for 72 h at 60°C, pooled and homogenised each species' individual modern leaves and individual historic leaves separately and then ground the dried leaf tissue. For each species, we sent a pooled sample of historic ground leaf tissue and a pooled sample of modern ground leaf tissue to the Environmental Analysis Laboratory at Southern Cross University, Lismore, Australia for nitrogen analysis following protocols in Rayment and Lyons (Rayment & Lyons, 2011).

### 2.3 Climate change metrics

Climate change metrics were determined for each species' historic and modern seed collection based geographically on modern seed collection site location data (which was collected typically at the same location as the historic data or within a 1 km radius, Everingham et al., 2021) and were obtained from the Australian Gridded Climate Data (Jones et al., 2009) at 5 km^2^ resolution following methods from Everingham et al. (2021). The processing code is freely available at https://github.com/SEveringham/ClimateData. The amount of change in all climate metrics was calculated across the 5 years before historic and modern seed collection to capture longer‐term climate change responses of the species without extending to a period of climate that may become non‐meaningful or overlap with modern climate data. The amount of change in precipitation metrics and heatwave duration were calculated using the log‐transformed ratio of means ($\ln\left(\frac{\text{modern climate metric}}{\text{historic climate metric}}\right)$). Change in all temperature metrics was calculated as the difference between the modern and historic climate metrics (modern climate metric−historic climate metric). We used different scaling methods because a difference of a few degrees Celsius of temperature has a much higher biological impact than a difference of a few millimetres of precipitation as precipitation has a much larger range of measurement than temperature. None of the climate change metrics was significantly correlated with one another (Everingham et al., 2021; all correlation coefficients were below 0.6) and therefore no climate metrics were excluded from our analyses.

The climate change metrics we used included the change between the modern and historic seed collections in mean monthly temperature (calculated as the daily median temperature in the month prior to the seed collection and averaged across the previous 5 years before the seed collection was made) and mean monthly precipitation (an average of precipitation from the month prior to seed collection and then averaged across the 5 years prior to collection). Both the change in the range of temperature and the range of precipitation were calculated as the change (between historic to modern collections) in the difference between the yearly maximum and minimum temperature or precipitation averaged across the 5 years prior to each seed collection. We also used metrics for change in temperature variability and change in precipitation variability, both of which were calculated as the coefficient of variation (standard deviation divided by the mean) of the temperature or precipitation of the month prior to seed collection averaged across the 5 years prior. The change in maximum and minimum precipitation of the season before collection were calculated to determine the effects of seasonal rainfall and these were an average across five prior years of collection of the maximum rainfall in the 4 months prior to seed collection (bound by wet season in the subtropics or autumn, winter, spring, summer seasons in the mid‐latitudes). We used the change in vapour pressure deficit (VPD) as an indicator of the change in atmospheric aridity between the historic and modern seed collections. VPD was determined from the difference in air moisture compared to moisture held at saturation. Finally, metrics of change in extreme climate events included the calculation of maximum heatwave duration (the longest heatwave across all seasons in the 5 years prior to collection, Nairn et al., 2009) and maximum dry spell duration (following the same protocol as maximum heatwave duration but instead with dry spells as calculated from an ‘extreme dryness index’ using VPD measurements).

### 2.4 Data analysis

We performed all data analysis in R, version 3.6.0 (R Core Team, 2020) with code freely available at https://github.com/SEveringham/leaf‐trait‐responses‐to‐climate‐change.

Change in traits or gas exchange variables was calculated for all morphological, photosynthetic and leaf economic traits or variables using the log‐transformed ratio of means per species ($\ln\left(\frac{\text{mean modern trait}}{\text{mean historic trait}}\right)$) using the *escalc* function in the *metafor* package (Viechtbauer, 2010). To determine whether the change in mean temperature or mean precipitation was related to any of the leaf morphological traits measured (roundness, margin complexity, surface area) or leaf physiology variables measured (photosynthetic rate, water use efficiency, stomatal density), we performed separate meta‐regressions using the *rma* function in the *metafor* package (Viechtbauer, 2010). In all models, the change in each leaf trait/gas exchange variable was the response variable (calculated as the log ratio of means), change in mean temperature or change in mean precipitation was a moderator (predictor) variable and models were weighted by sample variance (also calculated using the *escalc* function in the *metafor* package).

To determine if leaf economic spectra were related to changes in climate, we used Principal Components Analysis (PCA) to obtain metrics that combined the change in inverse LMA, photosynthetic rate and nitrogen content. The inverse of LMA (specific leaf area [SLA]) was used as it is negatively related to leaf economy (i.e. leaves that have a larger surface area per unit mass will have a lower LMA and are typically on the ‘faster’ end of the leaf economic spectrum). The PCA was achieved using the *prcomp* function in base R (R Core Team, 2020) and used imputed data as not all species had measurements for all three variables (imputation was done using the *imputePCA* function in the *missMDA* package (Josse & Husson, 2016)). The first principal component explained 82.06% of the variance and the second explained 17.94% of the variance of the change in the three leaf economic variables combined for each species (see Appendix S1: Table A4 for full results). A Horn's Parallel Analysis for component retention using the *paran* function in the *paran* package (Dinno, 2018) suggested that the retention of two components best explained the spread of the leaf economic trait data. We, therefore, regressed the two highest principal components against mean temperature and mean precipitation using the *rma* function in the *metafor* package (Viechtbauer, 2010). However, both components showed the same results and therefore, only the results from the first principal component that explained 82.06% of the variance are presented in our study (full results in Appendix S1: Tables A4 and A5).

Finally, to determine if changes in extreme climate metrics or climate variability metrics were more strongly related to changes in each leaf trait or variable than climatic means, we used a meta‐analytic model selection method with multi‐model variable inference using Akaike Information Criterion for small sample sizes (AICc; as our sample size ranged from 21 to 32 species for each leaf trait) using the functions *glmutli* and *coef* in the *glmulti* package (Calcagno & Mazancourt, 2010) and the *rma* function in the *metafor* package (Viechtbauer, 2010). This then allowed us to get the importance of each climate change variable on each leaf trait change variable averaged across all possible models in the model selection method (Burnham & Anderson, 2002). Our meta‐analytic models were built with the change in each leaf trait (calculated as the log‐transformed ratio of means, as above) as the response variable and all of the climate change metrics that were previously introduced in the methods as the moderator variables, as well as weighting term for sample variance.

### 2.5 Data considerations

Species used in this study to measure trait changes through time between historic and modern accessions were from a range of growth forms including four herbaceous species, ten shrub species, seven shrub‐tree species and eleven tree species (Figure 1; see Appendix S1: Table A1 for full species' growth‐form data). Species in these categories have different generation lengths and may respond to a larger or smaller extent in their leaf traits and variables to climate change over a given timeframe of 30–40 years. Generation time metrics and the data used to calculate species' generation times (including demographic and life‐history data) are generally scarce across many species, particularly in plants (Cooke et al., 2018; Staerk et al., 2019) and no data exist for the generation times of specific populations of the modern and historic seed collections in our study. We, therefore, used categorical growth form for all species (widely available data for all species in our study), age of reproductive maturity (data from AusTraits and NSW Flora Fire Response Database available for 15 out of 32 species in our study, Appendix S1: Table A1, Falster et al., 2021, Ferrer‐Paris & Keith, 2022) and average lifespan (data from AusTraits available for nine out of 32 species, Appendix S1: Table A1, Falster et al., 2021) as proxies for the average generation time of the species and populations in our study to determine whether generation time had any effect on the amount of change in the measured leaf traits and variables. A linear regression with plant growth‐form as a categorical predictor variable and the absolute average trait change across all leaf traits measured for each species as the response variable (log‐transformed due to non‐linearity) showed that there were no significant differences in the amount of average trait change occurring across species of differing growth‐forms (*R* ^2^ = −.01, *p* = .47) and this result reflects a similar non‐significant relationship found between growth‐form and the amount of change occurring in species' regeneration and growth traits (Everingham et al., 2021). Similarly, linear regressions with the average age of the species' reproductive maturity as a continuous predictor variable (*R* ^2^ = −.04, *p* = .49) and the average lifespan of our species as a continuous predictor variable (*R* ^2^ = −.11, *p* = .98) and the absolute average trait change across all leaf traits measured for each species as the response variable (log‐transformed due to non‐linearity) also showed no significant correlations. Species' growth‐form, age of reproductive maturity and lifespan were therefore not considered in any other analyses throughout the study.

Although we assumed the average atmospheric carbon dioxide (CO_2_) concentration change to have occurred equally across regions, there may be differences in the amount of CO_2_ change through time due to the different years of seed collection (see Appendix S1: Table A6 for seed collection dates). To determine whether the extent of changes in CO_2_ had an impact on the change in leaf traits or photosynthetic rates we regressed change in CO_2_ (calculated as the log‐transformed ratio of means) through time against change in leaf traits/variables (mean monthly atmospheric CO_2_ observations were obtained from the Mauna Loa observatory, NOAA ESRL (Tans & Keeling, 2017)). We found one significant relationship between change in CO_2_ and change in leaf photosynthetic rate, however, the magnitude of this change was low (Effect Size = −0.020, *R* ^2^ = .217, *p* = .008, see Appendix S1: Table A7 for full results). No other changes in leaf traits or variables were related to changes in CO_2_ so CO_2_ change was excluded from further analysis.

## 3 RESULTS

**FIGURE 2:**
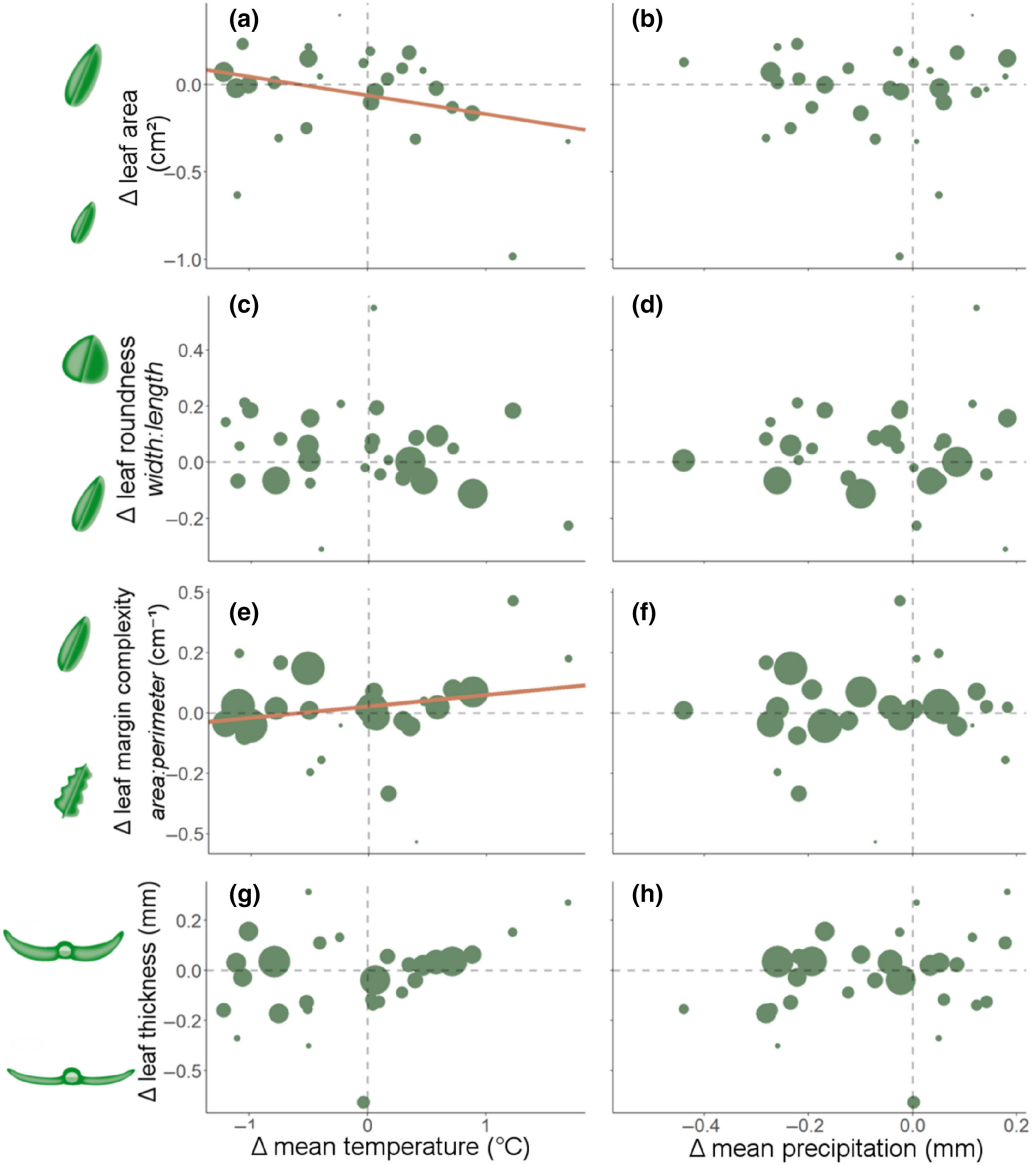
Panels a–h show relationships between change in (Δ) mean temperature (a, c, e, g) or change in mean precipitation (b, d, f, g) and change in four averaged leaf morphological metrics determined from pair‐wise meta‐analytic regressions. Each point represents the amount of change between historic and modern plants in one species calculated as the log‐transformed ratio of means per species. The confidence in the amount of trait change based on sampling variance is represented in the size of the points (larger points represent higher sample confidence). Significant relationships are represented by orange regression lines. Note that in (e) and (f) leaf margin complexity becomes more positive where leaves are less complex and have a higher area‐to‐perimeter ratio and more negative where leaves increase in complexity and have a lower area‐to‐perimeter ratio.

Contrary to our predictions, leaf surface area decreased as mean temperature increased, although this relationship was not statistically significant at α = 0.05 (*R* ^2^ = .086, *p* = .08; Figure 2a). For every 1°C increase in temperature, there was a decrease in leaf area by 1.7 mm^2^. However, the change in leaf area was not related to the change in mean precipitation (Figure 2b) and there were no other significant relationships between changes in leaf morphological traits (leaf margin complexity, leaf roundness and leaf thickness) and changes in mean temperature or mean precipitation (*p* > .05; Figure 2c–h, see Appendix S1: Table A8 for the full results). Leaf margin complexity did not show a significant relationship with mean temperature in a simple pairwise analysis, however, when analysed in a stepwise regression with multi‐model selection, leaf margin complexity increased significantly with increasing mean temperature (Figure 2e, variable importance = 99%, *R* ^2^ < .01, *p* < .001).

**FIGURE 3:**
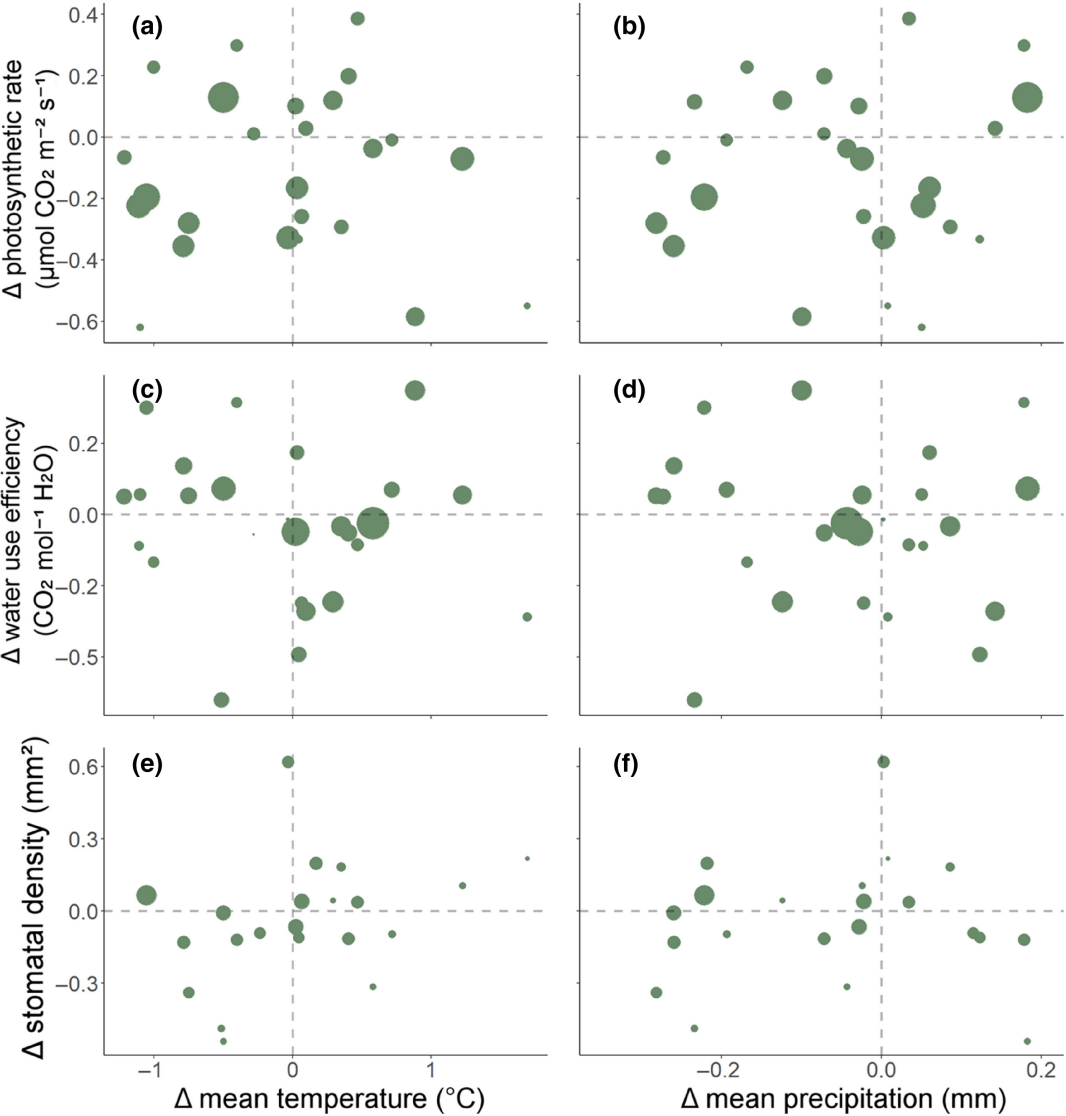
Panels a‐f show relationships between change in (Δ) mean temperature (a, c, e) and change in (Δ) mean precipitation (b, d, f) and change in (Δ) three averaged leaf photosynthetic variables, determined from pair‐wise meta‐analytic regressions. All relationships were non‐significant (*p* > .05). Each point represents the amount of change in one species between historic and modern plants calculated as the log‐transformed ratio of means per species. The confidence in the amount of trait change based on sampling variance is represented in the size of the points (larger points represent higher sample confidence).

Counter to our hypotheses, there were no significant relationships between the amount of change in any averaged photosynthetic variables (*A*_sat_, iWUE and stomatal density) and change in mean temperature or change in mean precipitation (*p* > .05, Figure 3, see Appendix S1: Table A8 for the full list of results).

**FIGURE 4:**
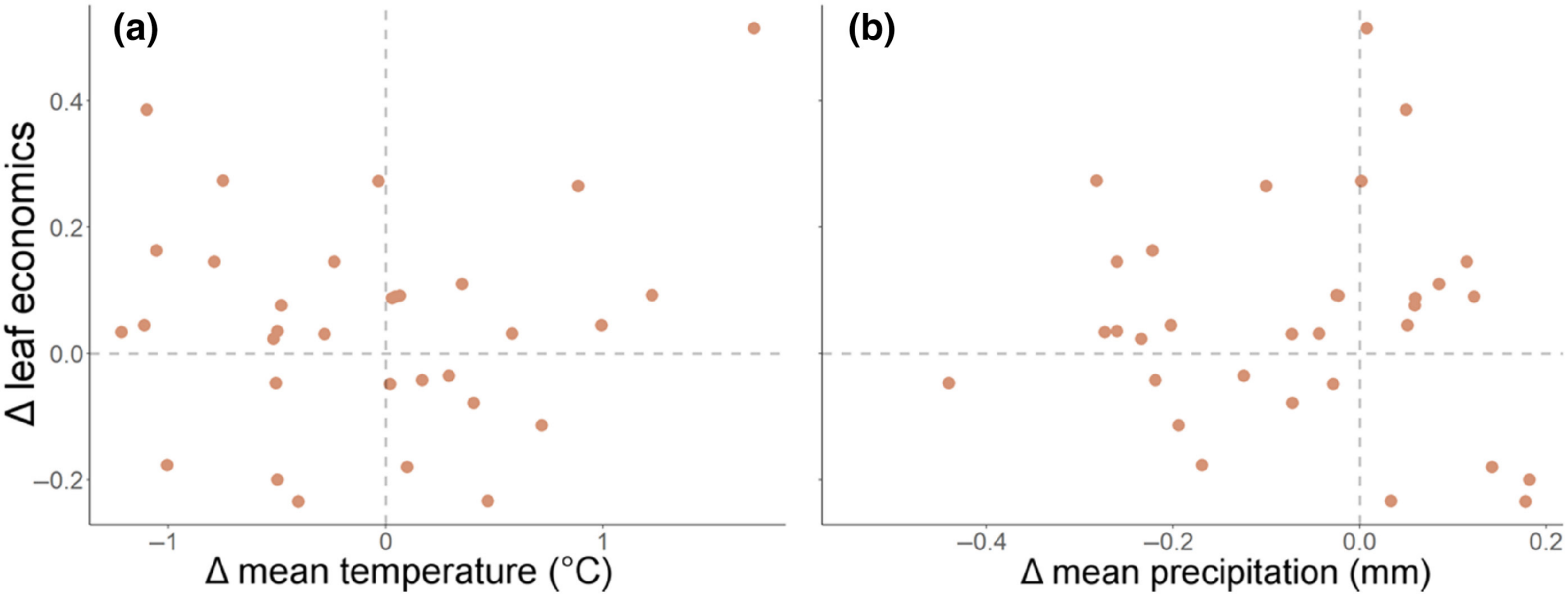
Relationship between change in mean temperature (a) and change in mean precipitation (b) and change in averaged leaf economics (calculated as a principal component from the change in photosynthetic rate, LMA and nitrogen content of leaves which were calculated from the log‐transformed ratio of means per species). Both relationships were non‐significant (*p* > .05). Each point represents the amount of change in leaf economics in one species between the modern and historic plants.

Change in leaf economic strategy (a metric calculated using PCA; see methods) was not significantly related to change in mean temperature (*R* ^2^ < .01, *p* = .908, Figure 4a) or change in mean precipitation (*R* ^2^ < .01, *p* = .947, Figure 4b).

At least one climate change metric was included in the best model (compared to the model with only the intercept) to explain changes in leaf physiology and morphology for half of the leaf traits and gas exchange variables. That is, changes in leaf area, leaf margin complexity, leaf thickness and iWUE are responding to changes in climate, and these three leaf traits are responding to a combination of changes in climate metrics rather than changes in a single climate metric (Table 1; excluding iWUE, where only one climate change variable—change in maximum drought duration—was selected). Our results show that leaf traits in our species are showing more responses to changes in climate variability and climate extremes than mean temperature and mean precipitation. Changes in mean temperature and mean precipitation were not always the strongest correlates of climate change. In fact, mean precipitation was only selected in one model (for change in leaf thickness). Changes in temperature variability, maximum and minimum seasonal precipitation, and climate extremes (including drought duration, heatwave duration and dry spell duration) were the climate change metrics that were most often selected in the best models that explained changes in leaf traits.

**TABLE 1:** Results from analysis of leaf morphological, physiological and leaf economic strategy changes about changes in climate metrics.

	Leaf area	Leaf roundness	Margin complexity	Leaf thickness	*A*_sat_	iWUE	Stomatal density	Leaf economic strategy
‘Best’ model results	*R* ^2^ = .619****	Intercept only	*R* ^2^ = .866***	*R* ^2^ = .443**	Intercept only	*R* ^2^ = .297**	Intercept only	Intercept only
Mean								
Temperature	0.76	0.37	0.98**	0.23	0.19	0.19	0.23	0.19
Precipitation	0.18	0.22	0.22	0.54	0.26	0.20	0.15	0.19
Variability								
Temperature	0.58	0.19	0.99****	0.19	0.18	0.16	0.24	0.19
Precipitation	0.21	0.19	0.50	0.21	0.35	0.18	0.20	0.19
Range								
Temperature	0.16	0.27	0.13	0.17	0.17	0.25	0.15	0.19
Precipitation	0.30	0.20	0.15	0.20	0.18	0.57	0.15	0.19
Seasonal precip.								
Max. precip of season	0.49	0.19	0.13	0.17	0.25	0.40	0.21	0.19
Min. precip of season	0.56	0.18	0.22	0.18	0.32	0.26	0.18	0.19
Aridity								
VPD	0.23	0.30	0.54	0.23	0.19	0.50	0.19	0.19
Climate extremes								
Max. drought duration	0.22	0.50	0.34	0.75	0.18	0.72	0.14	0.20
Max. heatwave duration	0.35	0.18	0.65	0.44	0.18	0.22	0.19	0.19
Max. dry spell duration	0.34	0.19	0.97**	0.18	0.17	0.16	0.42	0.19

*Note*: The top row of the table presents overall results from the ‘best’ model selected by AICc selection in a meta‐analytic framework. Cells shaded orange represent climate metrics selected in the best models using AICc stepwise model selection. Each cell indicates the importance of the various measures of climate in explaining the amount of leaf trait/variable and leaf economics changes. Importance is calculated as a proportion of each metric contributing to the trait change model that was calculated across all possible models in AICc model selection, using an average weighted value for each model. Overall model *R* ^2^ values are marginal *R* ^2^. Significance for best models and pairwise relationships are denoted in cells by asterisks (‘****’ for *p* < .0001, ‘***’ for *p* < .001, ‘**’ for *p* < .01).

**FIGURE 5:**
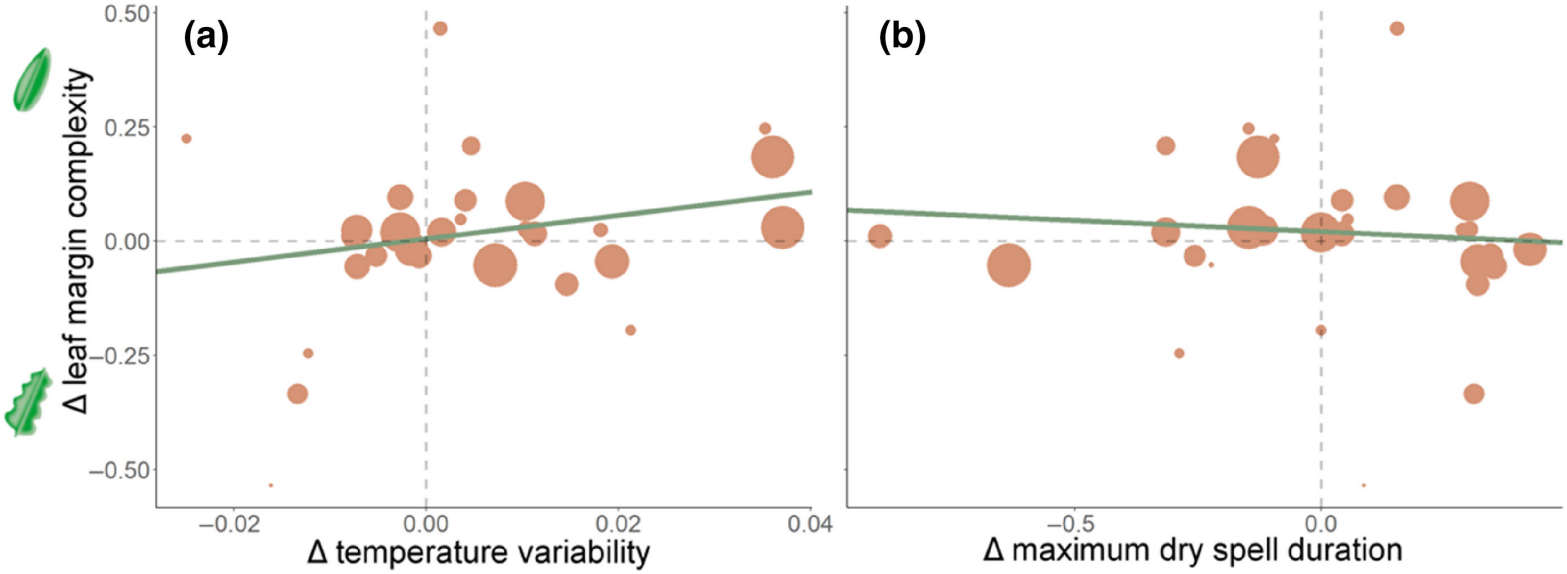
Relationships that were selected as significant across all possible models using AICc stepwise, multi‐model meta‐analytic regression. (a) change in temperature variability related to the change in leaf margin complexity and (b) change in maximum dry spell related to the change in leaf margin complexity. Change in mean temperature and change in margin complexity was also selected as a significant relationship and is depicted in Figure 2c. Each point represents the amount of change in one species between historic and modern plants. The confidence in the amount of trait or variable change based on sampling variance is represented in the size of the points (larger points represent higher sample confidence). Note that in (a) and (b) leaf margin complexity becomes more positive where leaves are less complex and have a higher area‐to‐perimeter ratio and more negative where leaves increase in complexity and have a lower area‐to‐perimeter ratio.

Across all possible models, the change in mean temperature and temperature variability were significant predictors of change in leaf margin complexity. Leaf margin complexity increased as mean temperature increased (Figure 2e, although this relationship was only found in multi‐model selection and not in the pairwise analysis, see results above). Leaf margin complexity increased as temperature variability increased (Table 1; Figure 5a) and decreased as the maximum duration of dry spells increased (Table 1; Figure 5b). Although these climate change metrics showed significant relationships with leaf margin complexity (*p* < .05), the magnitude of the change was low—for a 5% increase in temperature variability there was an increase of only 13% in leaf margin complexity. When there was an increase in dry spell duration of 1 day there was a 3% increase in leaf margin complexity (Figure 5).

## 4 DISCUSSION

We found that some leaf traits and variables are showing responses correlated with changes in climate over the last four decades (Figures 1 and 4; Table 1). This is positive news for plants' abilities to respond to future changes in climate as they may have the ability to respond rapidly and to survive changes in mean temperature and precipitation as well as climate variability and extremes. However, a large majority of changes in leaf traits, particularly physiological variables showed no correlation with climate changes. It is possible that this results from a lack of inherited adaptation to changes in the climate of origin of the seeds and/or to rapid acclimation to the growth conditions (since all the individual plants were exposed to the same growth and measurement conditions). Overall, we have been able to determine which particular leaf traits/variables are responding to which type of climate measures (i.e., climate means vs. extremes).

There may be concern about the potential for biases associated with seed storage that could affect the results of studies using the Resurrection Ecology approach. However, using our Climate Contrast Resurrection Ecology method (Everingham et al., 2021), we found that the intercepts for pairwise relationships of change in climate and change in plant traits were not significantly different to zero (*p* > .05, i.e. 0 on the *x*, *y* axes intercept in Figure 2 and Figure 5). That is, in the absence of climate changes, the species are not showing changes in leaf traits. This suggests that our study is not being substantially impacted by viability loss and storage bias of the historic seeds. A lack of changes in leaf traits may be because there are high levels of intraspecific trait variation within the modern and historic plant species which are not correlated with changes in climate through time. Although variation in the trait measurements is taken into consideration in our analyses (weighting the amount of change in trait or variable in each model by the variance in the trait measurements), large intraspecific trait variation within the modern plants and historic plants could mask overall significant relationships between leaf trait and leaf variable shifts and climate changes through time.

We had predicted that mean leaf area would increase in regions where temperatures had increased (consistent with evidence from studies across geographic gradients (Gallagher & Leishman, 2012b; Moles et al., 2014)). However, our results show that leaf area decreased as the mean temperature increased (Figure 2a). This may be an indication that species are reaching their upper extreme temperature limits and responding by reducing their leaf size. Reduction in leaf area in response to climate may be driven by genetic adaptation or epigenetic effects and future studies with more information and genetics from the parent populations (which is not available for the historic collections in our study) would be required to determine the mechanism behind this shift. Reduced leaf sizes may also lead to decreased light capture for photosynthesis, particularly if species leaf numbers do not increase rapidly enough as their leaf area decreases and this may lead to decreases in species' carbon acquisition and growth into the future.

Decreases in both leaf margin complexity (i.e. leaves becoming less lobed) and increases in leaf roundness should increase leaf boundary layer thickness, thus helping plants to cope with hot and dry conditions (Leigh et al., 2017). However, we found that only leaf margin complexity showed a significant response to changes in climate (leaf margins became less complex as mean temperature or temperature variability increased; Figures 2e and 5a respectively), while leaf roundness showed no significant response to any changes in climate (Figure 2; Table 1). Leaf margin complexity has been focused upon heavily in paleoclimatic studies and has shown to be more responsive to temperature than leaf roundness in broad geographic studies (Royer et al., 2005). It is well‐known that leaf margin complexity responds to long‐term climate change (Little et al., 2010; Royer & Wilf, 2006). However, our study now shows that this trait is also responsive to short‐term, anthropogenic climate change. Our study also provides a novel indication that there may be limited responses in leaf margin complexity to changes in precipitation, which was not previously quantified in paleoclimatic studies, however, geographic studies of leaf shape have shown that mean annual precipitation does not correlate with leaf roundness (Peppe et al., 2011). Decreases in leaf margin complexity due to climate change may have negative effects on other aspects of plant fitness such as carbon dioxide uptake.

Although plants have shown responses in their leaf morphology correlating with recent climate change (including shifts in leaf area and leaf margin complexity which showed significant relationships to climate change metrics, Figures 1 and 4; and the best model to explain leaf thickness included changes in climate extremes, Table 1), leaf physiological traits and variables showed far fewer responses to changes in climate metrics. In contrast to our predictions, no photosynthetic leaf traits or gas exchange variables (including photosynthetic rate, iWUE or stomatal density, Figure 3), nor leaf economic strategy (Figure 4), showed a significant inherent response to any changes in climate metrics (Table 1). Leaf economic strategy has shown to be weakly related, on broad geographic scales, to climate variables such as temperature and precipitation (Dwyer et al., 2014; Liu et al., 2013; Moles, 2018) and this is further supported by the lack of significant changes in leaf economic strategy in response to changes in climate in our study. Morphological traits may be more responsive or adaptive to anthropogenically‐induced changes in climate at longer time‐scale periods, i.e. over one or more years, decades or longer. Physiological traits may show fewer long‐term evolutionary responses and may be more responsive at a short‐term scale (over a few hours to days, or days to months), in a plastic mechanism (Cunningham & Read, 2002; Dewar et al., 1999). Future studies to determine the genetic mechanisms of the change would be required to disentangle whether physiological traits respond in clear changes between historic and modern populations over the long term or predominantly in a short‐term plastic response.

We found markedly fewer changes in leaf traits and variables in response to recent climate change than we found for regeneration and growth traits in a previous study using the same methods (Everingham et al., 2021). Manipulative experimental studies that quantified the effects of nutrient fertilisation (Funk et al., 2007) and drought (Monclus et al., 2006) on plants have found that plant growth traits show greater responses to environmental changes than plant leaf traits and variables. Biogeographic studies of plant traits have also determined that regeneration traits such as germination rate and seed dormancy have undergone greater responses than seedling traits (Dalgleish et al., 2010). Regeneration and growth traits could have the biggest impact on species fitness and survivability under climate change and environmental stress (Walck et al., 2010). Our study is the first to directly determine that in response to recent, anthropogenic climate change, leaf traits and gas exchange rates show minimal changes in comparison to plant traits such as regeneration and growth traits. Leaf traits may also be changing or responding non‐independently to changes in climate and future studies quantifying trait coordination within leaf traits and with other plant regeneration and growth traits will be required to determine if more complex responses involving a suite of traits are occurring in response to changes in climate.

Our results from pairwise analyses of leaf trait/variable responses to mean climate showed that species in our study responded more strongly in their leaf morphology traits to mean temperature than to mean precipitation (Figure 2). Change in mean precipitation was not selected in any of the best models (Table 1) and change in mean temperature was selected in the model that explained the greatest change in leaf area (Table 1) and leaf margin complexity (where it was a significant variable in the model to explain leaf margin complexity, *p* < .01, Table 1). Mean temperature tends to be more tightly correlated with plant traits globally than mean precipitation (Kloeke et al., 2012; Moles et al., 2014; Swenson & Enquist, 2007). Species may be less impacted by and respond less to mean precipitation, as changes in precipitation may not necessarily change the soil moisture content in the habitats of our species (Moles et al., 2014). Other factors such as soil type, soil depth and hydrology may affect species' water availability and may be mitigating low precipitation, thus leading to minimal or no changes in our species' leaf traits and photosynthetic rates through time in their natural habitats (Choat et al., 2007). In places where changes in temperature are expected to be more important than changes in precipitation, we can also expect the temperature to have greater impacts on plant morphological trait responses in the future.

Leaf traits and photosynthetic variables are showing stronger responses to climate variability and climate extremes than to climatic means (Table 1; Figure 4). While this might have been expected, based on the greater potential for selective mortality associated with changes in extreme climate (Jentsch & Beierkuhnlein, 2008), our study is the first to use Resurrection Ecology to determine which variables are the most important in driving morphological and physiological responses to climate change. Previous Resurrection Ecology research typically only focused on mean climate metrics (Dijk & Hautekèete, 2014) or one extreme climate metric alone (e.g. drought (Franks, 2011; Franks et al., 2007)). Changes in climate variability and extreme climate events may have a stronger effect on plant trait changes than more gradual changes in mean climate metrics as changes in climate variability and extremes pose novel conditions that plants have not adapted to and may not have been exposed to (Jentsch & Beierkuhnlein, 2008). In the future, extreme climate events are predicted to increase in frequency and duration and the climate is predicted to become more variable (Jentsch & Beierkuhnlein, 2008). Our results also indicated that leaf traits or variables typically responded to a combined set of climate change variables (Table 1), for example, a change in temperature with a change in seasonal precipitation led to a shift in leaf area. The interactive or additive effects of climatic variables may be leading to responses in leaf morphological traits in our species. It is vital to determine, measure and model not only species responses to mean climate change variables but also changes in extreme climate variables and climate variability, as well as the combination of these variables and how multiple climate drivers impact leaf trait change (Katz & Brown, 1992).

Using an emerging resource—resurrected plants—we were able to directly measure proxies of historic plant physiological traits and gas exchange variables that were not measured in the past. In addition to enabling researchers to study previously unmeasured traits and variables, the Climate Contrast Resurrection Ecology approach (Everingham et al., 2021) could be applied to seed collections in museums and ex‐situ seed banks (e.g. Kew Botanic Gardens Millennium Seed Bank) for future studies to estimate responses to climate change in species for which historical analogue data are not available. This could include understudied taxa and species from understudied regions (e.g. a range of areas in the southern hemisphere). Global collaborations such as *Project Baseline* (Etterson et al., 2016) will also benefit from the Climate Contrast Resurrection Ecology method as they begin to store seeds in the current day to determine plant trait responses and adaptations to climate change in the future.

In summary, our three main findings were: (1) some leaf traits in seedlings have shown responses to climate change, (2) morphological traits are responding more to changes in climate than are physiological traits and variables and (3) leaf traits and variables are responding more to changes in extreme measures of climate and climate variability than changes in mean climate metrics. Overall, our results indicate that plants may be able to respond to and therefore adapt to changes in mean temperature and respond and survive under the current rate of increase in extreme events. However, many traits that contribute to plant fitness/survival such as photosynthesis and water use efficiency are showing limited responses to climate change and therefore may impede species survival under future climate change especially as the rate of change continues to increase (Collins et al., 2013). Understanding which traits are responding to climate change is essential for future climatic ecological studies.

## AUTHOR CONTRIBUTIONS

**Susan E. Everingham:** Data curation (lead); formal analysis (lead); funding acquisition (equal); investigation (equal); methodology (equal); project administration (equal); visualization (lead); writing – original draft (lead); writing – review and editing (lead). **Catherine A. Offord:** Data curation (supporting); investigation (equal); methodology (equal); resources (equal); writing – original draft (equal); writing – review and editing (equal). **Manon E. B. Sabot:** Data curation (equal); formal analysis (equal); funding acquisition (equal); methodology (equal); writing – original draft (equal); writing – review and editing (equal). **Angela T. Moles:** Conceptualization (lead); data curation (supporting); formal analysis (supporting); funding acquisition (equal); investigation (equal); methodology (equal); resources (equal); supervision (lead); visualization (supporting); writing – original draft (equal); writing – review and editing (equal).

## Supporting information


Appendix S1

## Data Availability

Upon publication of the manuscript, the authors will make all data publicly available on Dryad. To access the private repository for peer review please follow the link: https://datadryad.org/stash/share/5uBVZjl1zgLWFe9ue6ziCNyM9N7G0GZ05TlRRB6FIPA.
