# Supplementary material for: Leaf morphological traits show greater responses to changes in climate than leaf physiological traits and gas exchange variables
Source: Ecol Evol. 2024 Mar 19;14(3):e10941. doi: 10.1002/ece3.10941 (PMC10951557; doi:10.1002/ece3.10941)
Supplement: Supplementary file 1 — Appendix S1 [file ECE3-14-e10941-s001.docx]

**Appendix A**

**Table A1.** Full species list, growth form, reproductive maturity, lifespan, leaf type and collection location from study

**Section A1.** Detailed protocol on germination of species’ modern and historic seeds.

**Table A2.** Germination treatments for all species seeds used in study

**Section A2.** Detailed protocol and sample sizes for leaf trait measurements

**Table A3.** Sample sizes for photosynthetic measurements

**Table A4.** Detailed results of leaf economics PCA analysis

**Table A5.** Full results of meta-analytic multi-model selection with variable inference for the second principal component of the leaf economic principal components analysis

**Figure A1.** Relationship between change in mean temperature (a) and change in mean precipitation (b) and change in leaf economics

**Table A6.** Seed collection dates for historic and modern species

**Table A7.** Results of leaf trait changes responses to CO_2_

**Table A8.** Complete analyses results for trait change responses to changes in mean temperature and precipitation

**Table A1.** Full species list, growth form, reproductive maturity, lifespan, leaf type and collection location from study

| Species | Growth form | Average age  of reproductive  maturity in years  (data from  AusTraits*^+^) | Average lifespan  (data from AusTraits) | Leaf type (for *Acacia*  species this is specified  for adult leaves only) | Collection Location  (deg min sec) | |
| --- | --- | --- | --- | --- | --- | --- |
|  |  |  |  |  | **Latitude** | **Longitude** |
| *Acacia anceps* | Shrub | NA | NA | Simple | 33 43 40 S | 135 51 25 E |
| *Acacia aspera* | Shrub | NA | NA | Simple | 34 32 38 S | 147 43 49 E |
| *Acacia baileyana* | Tree | 3.33 | 25 | Compound | 34 33 22 S | 147 43 49 E |
| *Acacia brachybotrya* | Shrub | 3.5^+^ | NA | Simple | 32 47 43 S | 134 12 40 E |
| *Acacia chinchillensis* | Shrub | NA | NA | Compound | 26 45 19 S | 150 37 42 E |
| *Acacia concurrens* | Shrub-tree | NA | NA | Simple | 26 38 60 S | 153 04 00 E |
| *Acacia cultriformis* | Shrub | NA | NA | Simple | 29 00 59 S | 151 30 14 E |
| *Acacia dealbata* | Shrub-tree | 5.2 | 20 | Compound | 42 16 55 S | 146 37 09 E |
| *Acacia deanei* | Shrub-tree | NA | NA | Compound | 34 44 07 S | 146 34 11 E |
| *Acacia georgensis* | Shrub-tree | NA | 25 | Simple | 36 39 34 S | 149 54 20 E |
| *Acacia hakeiodes* (Bourke) | Shrub-tree | NA | NA | Simple | 30 28 54 S | 149 36 19 E |
| *Acacia hakeiodes* (Temora) | Shrub-tree | NA | NA | Simple | 34 15 43 S | 146 10 08 E |
| *Acacia mariae* | Shrub | NA | NA | Simple | 30 34 36 S | 149 26 52 E |
| *Allocasuarina littoralis* | Tree | 6.5 | 55 | Modified branchlets with reduced leaves | 29 07 26 S | 153 25 56 E |
| *Allocasuarina nana* | Shrub | 10.0 | 75 | Modified branchlets with reduced leaves | 33 38 38 S | 150 14 42 E |
| *Bothriochloa macra* | Herb | 1.0 | NA | Simple | 35 21 30 S | 149 12 53 E |
| *Bulbine bulbosa* | Herb | 1.5 | NA | Simple | 35 28 25 S | 149 0 47 E |
| *Callistemon sieberi* | Shrub-tree | 4.0 | NA | Simple | 35 19 47 S | 149 15 06 E |
| *Callitris endlicheri* | Tree | 7.0 | NA | Simple | 35 19 50 S | 149 15 40 E |
| *Corymbia eximia* | Tree | NA | 100 | Simple | 33 27 23 S | 150 43 05 E |
| *Corymbia gummifera* | Tree | 3.0 | 250 | Simple | 33 27 23 S | 150 43 05 E |
| *Eucalyptus calcareana* | Tree | NA | NA | Simple | 31 57 28 S | 132 23 4 E |
| *Eucalyptus crebra* | Tree | NA | 100 | Simple | 29 05 36 S | 151 46 51 E |
| *Eucalyptus globoidea* | Tree | NA | 100 | Simple | 37 25 20 S | 149 11 53 E |
| *Eucalyptus nicholii* | Tree | NA | NA | Simple | 30 28 52 S | 152 17 34 E |
| *Eucalyptus obliqua* | Tree | 2.0 | 100 | Simple | 42 18 51 S | 147 54 06 E |
| *Hovea lanceolata* | Shrub | 4.0 | NA | Simple | 29 05 36 S | 151 46 51 E |
| *Lasiopetalum behrii* | Shrub | NA | NA | Simple | 34 45 15 S | 135 47 32 E |
| *Melaleuca gibbosa* | Shrub | NA | NA | Simple | 43 18 17 S | 147 17 47 E |
| *Melaleuca quinquenervia* | Tree | 3.0 | 100 | Simple | 31 33 01 S | 152 51 05 E |
| *Senecio garlandii* | Herb | 2.5 | NA | Simple | 35 16 28 S | 147 04 34 E |
| *Vittadinia cuneata* | Herb | 1.0* | NA | Simple | 35 22 24 S | 149 12 47 E |

***** One species datapoint – *Vittadinia cuneata* for time to reproductive maturity from NSW, Flora Fire Response Database. ^+^ One species datapoint – *Acacia brachybotrya* for time to reproductive maturity from personal communication from Professor Ross Bradstock (NSW, Australia Fire Ecologist).

**Section A1.** Detailed protocol on germination of species’ modern and historic seeds.

Most species were germinated at 20°C with a 12-hour light, 12-hour dark cycle, but some species required specific germination treatments such as gibberellic acid (GA_3_), smoke water (1%) or specific temperature and light treatments (see Table S1 for full germination treatment methods). Treatments were kept constant between historic seeds and modern seeds.

**Table A2**: detailed conditions used for germinating each species’ modern and historic seeds. All seeds experienced a cycle of 12hr light and 12hr dark conditions. GA_3_ is gibberellic acid used in the agar substrate.

| Species | Seeds  Scarified (yes/no) | Germination  substrate | Germination temperature |
| --- | --- | --- | --- |
| *Acacia anceps* | Y | Water agar 0.7% | 20^°^C |
| *Acacia aspera* | Y | Water agar 0.7% | 20^°^C |
| *Acacia baileyana* | Y | Water agar 0.7% | 20^°^C |
| *Acacia brachybotrya* | Y | Water agar 0.7% | 20^°^C |
| *Acacia chinchillensis* | Y | Water agar 0.7% | 20^°^C |
| *Acacia concurrens* | Y | Water agar 0.7% | 20^°^C |
| *Acacia cultriformis* | Y | Water agar 0.7% | 20^°^C |
| *Acacia dealbata* | Y | Water agar 0.7% | 20^°^C |
| *Acacia deanei* | Y | Water agar 0.7% | 20^°^C |
| *Acacia georgensis* | Y | Water agar 0.7% | 20^°^C |
| *Acacia hakeiodes* (Bourke) | Y | Water agar 0.7% | 20^°^C |
| *Acacia hakeiodes* (Temora) | Y | Water agar 0.7% | 20^°^C |
| *Acacia mariae* | Y | Water agar 0.7% | 20^°^C |
| *Allocasuarina littoralis* | N | Water agar 0.7% | 20^°^C |
| *Allocasuarina nana* | N | Water agar 0.7%, GA_3_ | 20^°^C |
| *Bothriochloa macra* | N | Water agar 0.7% | 20^°^C |
| *Bulbine bulbosa* | N | Water agar 0.7%, GA_3_ | 20^°^C |
| *Callistemon sieberi* | N | Water agar 0.7%, GA_3_ | 20^°^C |
| *Callitris endlicheri* | N | Water agar 0.7% | 20^°^C |
| *Corymbia eximia* | N | Water agar 0.7% | 20^°^C |
| *Corymbia gummifera* | N | Water agar 0.7% | 20^°^C |
| *Eucalyptus calcareana* | N | Water agar 0.7% | 20^°^C |
| *Eucalyptus crebra* | N | Water agar 0.7% | 20^°^C |
| *Eucalyptus globoidea* | N | Water agar 0.7% | 20^°^C |
| *Eucalyptus nicholii* | N | Water agar 0.7% | 20^°^C |
| *Eucalyptus obliqua* | N | Water agar 0.7% | 20^°^C |
| *Hovea lanceolata* | Y | Water agar 0.7% | 20^°^C |
| *Lasiopetalum behrii* | Y | Water agar 0.7% | 20^°^C |
| *Melaleuca gibbosa* | N | Water agar 0.7% | 20^°^C |
| *Melaleuca quinquenervia* | N | Water agar 0.7% | 20^°^C |
| *Senecio garlandii* | N | Water agar 0.7% | 20^°^C |
| *Vittadinia cuneata* | N | Water agar 0.7%, GA_3_ | 20^°^C |

**Section A2.** Detailed protocol and sample sizes for leaf trait measurements

Leaf trait measurements on seedlings follow protocols from Pérez-Harguindeguy (2013).

To measure leaf shape, leaf area and leaf mass per unit area (LMA), we collected three fresh leaves (excluding the petiole) from each individual plant at the end of the six-month growing period. For two species (*Acacia georgensis* and *Acacia concurrens*), due to their seedling size, we were not able to measure area on three leaves and one to two leaves were sampled. Images of these fresh leaves were captured on a Flatbed Scanner and their area and shape metrics were calculated using values measured in image analysis software, ImageJ (Schneider et al., 2012).

Leaf surface area was calculated as the average of the three leaves’ total surface area.

ImageJ provided a measurement for each leaf of the maximum length (longest axis of the smallest possible rectangle drawn around the leaf) and width (longest axis perpendicular to the determined maximum length). From these measurements we calculated leaf roundness as the average ratio of width to length of the three leaves whereby the leaves with roundness measurements closer to zero would be longer, thinner leaves and the leaves closer or equal to 1 would be rounder leaves.

We calculated the margin complexity as the average of the ratio of perimeter length (cm) to surface area (cm^2^) from the perimeter of the leaf and the area analysed in ImageJ.

To calculate leaf mass per unit area we used the leaf surface area calculations measured in ImageJ. The leaves were then dried to a constant temperature using a drying oven at 60° C for 72 hr. Oven dry mass (g) for the leaves was measured by weighing on a microbalance (Mettler Toledo© *AG204 microbalance*, 1 x 10^-4^ accuracy). LMA was calculated as oven-dry mass divided by fresh area.

We measured leaf thickness by sampling one leaf from each individual modern and historic plant from all species (the third leaf from the growing tip, counted from the first fully developed/unfolded leaf). On these leaves we measured fresh leaf thickness (mm) at two points on adjacent sides of the mid-vein using a micrometer. An average for leaf thickness was taken from the two measurements for each individual plant.

Finally, we calculated stomatal density using the clear nail polish peel method (Wyers & Travis, 2005. Clear nail polish peels were performed on the first mature leaf closest to the growing apical tip from each plant. Clear nail polish was painted on the top and underside of the leaf on fresh tissue, away from the mid-vein or any prominent veins. We allowed the nail polish to dry for approximately 60 seconds before removing and mounting on a microscope slide with a coverslip. The peels were then imaged using a Leica© microscope. Stomata in each image were counted manually for the top of the leaf and the bottom of the leaf and the average stomatal density (stomata.cm^-2^) was calculated for each plant and use in further analysis.

**References**

Pérez-Harguindeguy, N. et al. (2013) New handbook for standardised measurement of plant functional traits worldwide. *Australian Journal of Botany*, *61*, 167–70. doi:10.1071/BT12225

Schneider, C. A., Rasband, W. S. & Eliceiri, K. W. (2012) NIH Image to ImageJ: 25 years of image analysis. *Nature Methods*, 1–5. doi:10.1038/nmeth.2089

Weyers, J. D. B. & Travis, A. J. (2005) Selection and Preparation of Leaf Epidermis for Experiments on Stomatal Physiology*. Journal of Experimental Botany*, 32, 837–850.

**Table A3.** Sample sizes for modern and historic plant photosynthetic rate (A_sat_) and intrinsic water use efficiency (iWUE) measured in study. One leaf per sample was measured at five consecutive intervals using a Licor6400 machine. Where there were no samples, species did not have enough tissue to be measured and they are not included in the gas exchange analyses in our study.

| Species | Sample size for gas exchange measurements on Licor6400 | |
| --- | --- | --- |
|  | Modern | Historic |
| *Acacia anceps* | 10 | 10 |
| *Acacia aspera* | 2 | 4 |
| *Acacia baileyana* | 10 | 10 |
| *Acacia brachybotrya* | 10 | 10 |
| *Acacia chinchillensis* | 0 | 0 |
| *Acacia concurrens* | 0 | 0 |
| *Acacia cultriformis* | 10 | 10 |
| *Acacia dealbata* | 9 | 5 |
| *Acacia deanei* | 10 | 10 |
| *Acacia georgensis* | 0 | 0 |
| *Acacia hakeiodes* (Bourke) | 10 | 10 |
| *Acacia hakeiodes* (Temora) | 10 | 6 |
| *Acacia mariae* | 10 | 10 |
| *Allocasuarina littoralis* | 10 | 4 |
| *Allocasuarina nana* | 0 | 0 |
| *Bothriochloa macra* | 10 | 3 |
| *Bulbine bulbosa* | 10 | 10 |
| *Callistemon sieberi* | 9 | 10 |
| *Callitris endlicheri* | 10 | 6 |
| *Corymbia eximia* | 10 | 10 |
| *Corymbia gummifera* | 10 | 10 |
| *Eucalyptus calcareana* | 10 | 10 |
| *Eucalyptus crebra* | 10 | 10 |
| *Eucalyptus globoidea* | 3 | 3 |
| *Eucalyptus nicholii* | 0 | 0 |
| *Eucalyptus obliqua* | 10 | 10 |
| *Hovea lanceolata* | 10 | 6 |
| *Lasiopetalum behrii* | 4 | 6 |
| *Melaleuca gibbosa* | 10 | 10 |
| *Melaleuca quinquenervia* | 10 | 4 |
| *Senecio garlandii* | 10 | 6 |
| *Vittadinia cuneata* | 10 | 10 |

**Table A4.** Results from the PCA to combine three metrics of leaf economic trade off (LMA, photosynthetic rate, nitrogen) to obtain one metric that explained the highest amount of species leaf economy.

|  | PC1 | PC2 | PC3 |
| --- | --- | --- | --- |
| Standard deviation | 1.0177 | 0.4759 | 8.645 x10^-17^ |
| Proportion of variance | 0.8206 | 0.1794 | <0.0001 |
| Cumulative proportion | 0.8206 | 1.0000 | 1.0000 |

**Table A5.** Full results of meta-analytic multi-model selection with variable inference for the second principal component of the leaf economic principal components analysis which explained 17.94% of the variance in the data but was retained due to a Horn’s Parallel Analysis for component retention.

|  | |  | *Principal Component 2 for Leaf Economics* |
| --- | --- | --- | --- |
| *“Best” model results* | | | No Model Selected |
| *Mean* | Temperature | | Estimate: 0.005, p = 0.934 |
|  | Precipitation | | Estimate: 0.019, p = 0.941 |
| *Variability* | Temperature | | Estimate: -0.254, p = 0.926 |
|  | Precipitation | | Estimate: 0.038, p = 0.926 |
| *Range* | Temperature | | Estimate: 0.005, p = 0.780 |
|  | Precipitation | | Estimate: -0.001, p = 0.989 |
| *Seasonal precip.* | Max. precip of season | | Estimate: 0.025, p = 0.835 |
|  | Min. precip of season | | Estimate: -0.011, p = 0.952 |
| *Aridity* | VPD | | Estimate: 0.031, p = 0.960 |
| *Climate extremes* | Max. drought duration | | Estimate: -0.029, p = 0.859 |
|  | Max. heatwave duration | | Estimate: 0.001, p = 0.927 |
|  | Max. dry spell duration | | Estimate: -0.012, p = 0.927 |

**
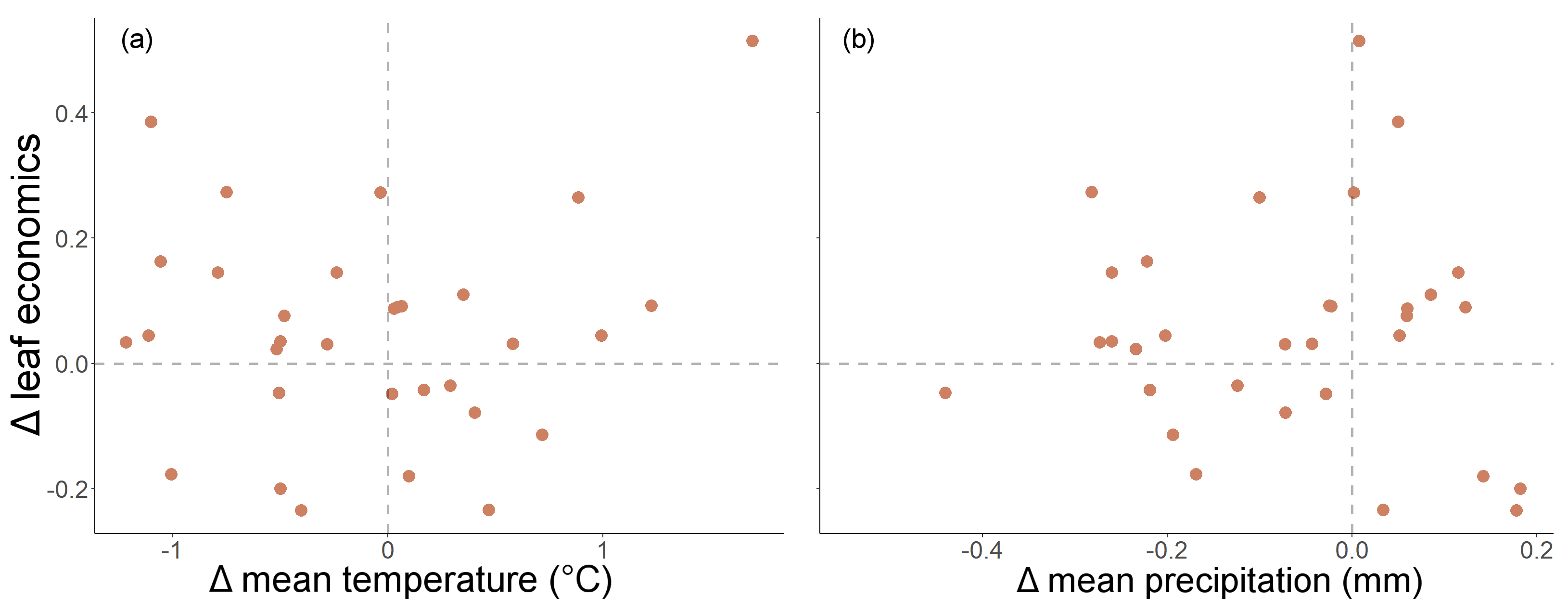
**

**Figure A1:** relationship between change in mean temperature (a) and change in mean precipitation (b) and change in leaf economics (calculated as a principal component from photosynthetic rate, LMA and nitrogen content of leaves). Both relationships were non-significant (p > 0.05). Each point represents the amount of change in leaf economics in one species between the modern and historic plants.

**Table A6.** Collection date for historic and modern seed accessions. Four species only had collection data to a month resolution and are indicated as Month-Year.

| Species | Historic seed collection date  (dd/mm/yyyy) | Modern seed collection date  (dd/mm/yyyy) |
| --- | --- | --- |
| *Acacia anceps* | 27/11/1985 | Dec-2016 |
| *Acacia aspera* | 24/11/1984 | 24/11/2017 |
| *Acacia baileyana* | 24/11/1984 | 24/11/2017 |
| *Acacia brachybotrya* | 27/11/1985 | Nov-2016 |
| *Acacia chinchillensis* | 27/11/1984 | 1/09/2009 |
| *Acacia concurrens* | 25/11/1984 | 1/12/2016 |
| *Acacia cultriformis* | 22/11/1984 | 6/11/2017 |
| *Acacia dealbata* | 25/02/1986 | 26/01/2018 |
| *Acacia deanei* | 27/06/1984 | 23/11/2017 |
| *Acacia georgensis* | 14/12/1984 | 20/12/2016 |
| *Acacia hakeiodes* (Bourke) | 2/12/1984 | 5/11/2017 |
| *Acacia hakeiodes* (Temora) | 27/11/1984 | 23/11/2017 |
| *Acacia mariae* | 2/12/1984 | 4/10/2017 |
| *Allocasuarina littoralis* | 26/03/1982 | 8/11/2017 |
| *Allocasuarina nana* | 13/07/1988 | 17/08/2017 |
| *Bothriochloa macra* | 15/04/1980 | 11/12/2017 |
| *Bulbine bulbosa* | 10/12/1980 | 1/12/2016 |
| *Callistemon sieberi* | 23/05/1980 | 11/12/2017 |
| *Callitris endlicheri* | 15/01/1979 | 11/12/2017 |
| *Corymbia eximia* | 10/02/1983 | 26/02/2018 |
| *Corymbia gummifera* | 21/01/1986 | 27/02/2018 |
| *Eucalyptus calcareana* | 20/10/1985 | 13/11/2017 |
| *Eucalyptus crebra* | 2/11/1983 | 6/11/2017 |
| *Eucalyptus globoidea* | 26/10/1977 | 13/12/2017 |
| *Eucalyptus nicholii* | 22/04/1986 | 12/01/2017 |
| *Eucalyptus obliqua* | 1/03/1986 | 24/01/2018 |
| *Hovea lanceolata* | 22/11/1984 | Nov-2008 |
| *Lasiopetalum behrii* | 27/11/1985 | Nov-2014 |
| *Melaleuca gibbosa* | 29/12/1979 | 18/01/2018 |
| *Melaleuca quinquenervia* | 21/10/1978 | 17/10/2017 |
| *Senecio garlandii* | 26/11/1984 | 22/11/2017 |
| *Vittadinia cuneata* | 18/12/1986 | 12/12/2017 |

**Table A7.** Results from linear regression analyses with change in atmospheric CO_2_ data downloaded from the ESRL NOAA datasets collected at Mauna Loa, Hawaii as the predictor variable and each leaf trait change as the response variable.

| Plant trait change | Estimate | R^2^ | p-value |
| --- | --- | --- | --- |
| Leaf area | -0.001 | <0.01 | 0.839 |
| Leaf roundness | -0.005 | 0.15 | 0.073 |
| Leaf margin complexity | 0.003 | <0.01 | 0.379 |
| Leaf thickness | -0.002 | <0.01 | 0.700 |
| Photosynthetic rate | -0.020 | 0.22 | 0.008* |
| Water use efficiency | 0.002 | <0.01 | 0.842 |
| Stomatal density | 0.006 | <0.01 | 0.681 |
| Leaf economics | 0.010 | <0.01 | 0.691 |

**Table A8.** Complete analyses results for leaf trait changes in response to changes in mean temperature and precipitation

|  | Temperature | | | Precipitation | | |
| --- | --- | --- | --- | --- | --- | --- |
|  | *Estimate* | *R^2^* | *p-value* | *Estimate* | *R^2^* | *p-value* |
| Leaf area | -0.107 | 0.086 | 0.082* | -0.039 | 0.019 | 0.120 |
| Leaf roundness | 0.045 | <0.001 | 0.745 | 0.047 | <0.001 | 0.186 |
| Leaf margin complexity | 0.081 | 0.001 | 0.618 | 0.071 | <0.001 | 0.155 |
| Leaf thickness | 0.071 | <0.001 | 0.155 | 0.242 | <0.001 | 0.280 |
| Photosynthetic rate | -0.015 | <0.001 | 0.828 | 0.180 | <0.001 | 0.622 |
| Water use efficiency | -0.044 | <0.001 | 0.548 | -0.138 | <0.001 | 0.724 |
| Stomatal density | 0.115 | 0.008 | 0.195 | 0.115 | <0.001 | 0.750 |
| Leaf economics | 0.029 | <0.001 | 0.908 | -0.075 | <0.001 | 0.947 |
